# Vaccine Hesitancy in the Autism Spectrum Disorder Context: Parental Vaccine Decision-Making and Coping with Stress Strategies

**DOI:** 10.1007/s10803-024-06508-x

**Published:** 2024-08-08

**Authors:** Bugra Taygun Gulle, Ugur Yassibas, Enes Sarigedik

**Affiliations:** 1 Faculty of Medicine, Department of Public Health, Devision of Epidemiology, Dokuz Eylül University, 35330-Balcova İzmir, Izmir, Turkey; 2 Department of Special Education, Sakarya University Faculty of Education, Sakarya, Turkey; 3 Faculty of Medicine, Department of Child and Adolescent Psychiatry, Sakarya University, Sakarya, Turkey

**Keywords:** Autism Spectrum Disorder, Vaccine Hesitancy, Coping Mechanisms, Neurodevelopmental Disorders

## Abstract

**Background:**

Despite no scientific evidence linking vaccines to Autism Spectrum Disorder (ASD), vaccine hesitancy persists among parents of children with ASD. This study aims to compare vaccine hesitancy and behaviors among parents of children with ASD, other Neurodevelopmental Disorders (NDD), and without NDD, and to examine the relationship between stress coping mechanisms and vaccine hesitancy, including comparing coping mechanisms between diagnostic groups as well as their association with hesitancy.

**Methods:**

In this cross-sectional study, one parent of each child with ASD, non-ASD NDD, or without NDD was included. Data were collected using a researcher-created form, the Vaccine Hesitancy Scale in Turkish, and the Coping Style Scale Brief Form. Vaccine hesitancy, parents’ COVID-19 vaccination status, and vaccination status of children’s younger siblings were analyzed through univariate and multivariate analyses, with a focus on correlations between vaccine hesitancy and coping styles.

**Results:**

The study included one parent from each of 299 children. Parents of children with ASD showed an adjusted odds ratio of 2.66 (95% CI 1.35–5.06) for high vaccine hesitancy, 2.57 (95% CI 1.17–5.65) for not receiving the COVID-19 vaccine, and 1.40 (95% CI 0.45–4.40) for younger siblings not receiving routine vaccines. A weak but significant correlation was observed between vaccine hesitancy and the use of restraint coping style among these parents (*r* = 0.280; *p* = 0.010).

**Conclusions:**

The study underscores the importance of targeted educational efforts and personalized communication to address vaccine hesitancy among parents of children with ASD. Enhancing vaccination coverage in this community requires further research to develop interventions tailored to their specific needs.

## Introduction

It is widely acknowledged that vaccination is considered one of the greatest achievements in public health (Centers for Disease Control and Prevention (CDC), 1999, 2011). Vaccination has been crucial in eradicating diseases like smallpox and now prevents over 20 diseases, saving an estimated 3.5-5 million lives annually (Andre, 2003; World Health Organization, 2022). Despite these remarkable achievements in the field of vaccination, vaccine hesitancy remains a significant global health concern (World Health Organization, 2019).

Vaccine hesitancy encompasses a diverse spectrum of attitudes, ranging from unwavering acceptance of all vaccines to outright rejection (MacDonald, 2015). High levels of vaccine hesitancy among parents can result in delays in childhood vaccinations or lead to certain vaccinations not being administered (Sahni et al., 2020). Vaccine hesitancy can become an even more significant issue in populations such as families of children with Autism Spectrum Disorder (ASD), due to increased concerns about the perceived risks of vaccination potentially exacerbating ASD symptoms (Bonsu et al., 2021; Goin-Kochel et al., 2020).

ASD is defined as “being social communication deficits and repetitive and unusual sensory motor behaviours” a description that has not changed substantially since its original delineation (Lord et al., 2018). Approximately one in every 36 children in the United States has been identified with ASD (Centers for Disease Control and Prevention (CDC). 2024). In Türkiye, however, the prevalence of ASD is not well-documented (Susuz and Doğan 2020). The etiology of ASD is unknown for the majority of cases, which may be one of the factors leading to the perception of an association between ASD and vaccines (Zerbo et al., 2018).

After the publication by Wakefield et al. which initially suggested a link between ASD and the measles, mumps, and rubella (MMR) vaccine, but was later retracted, numerous subsequent studies have consistently refuted any such association (DeStefano & Shimabukuro, 2019; Gidengil et al., 2021; Taylor et al., 2014; Wakefield et al., 1998). Nevertheless, it has been observed that some parents still perceive vaccines as a potential cause of ASD and believe that specific vaccines may be linked to the development of ASD. (Goin-Kochel et al., 2015, 2020; Mohamed et al., 2019; Pivetti et al., 2020; Sahni et al., 2020). Consequently, parents often exhibit a heightened degree of skepticism when it comes to vaccinating their children with ASD or younger siblings of children with ASD, resulting in lower vaccination (Bağ & Güney, 2023; Basheer & Uvais, 2022; Bayturan & Celasin, 2022; Rosenberg et al., 2013; Zerbo et al., 2018). Additionally, these individuals may also be more hesitant about getting adult vaccinations for themselves, such as the COVID-19 vaccine (Al Saad et al., 2023; Khoodoruth et al., 2023).

The majority of studies evaluating vaccine acceptance have been conducted among parents, mainly because most vaccines are aimed at children and adolescents (Dubé et. al. 2013). Research often centers on the vaccination status of children with ASD and their siblings, parental vaccine hesitancy and its impact on children’s vaccination status is often overlooked. Moreover, few studies compare hesitancy levels between parents of children with ASD, other Neurodevelopmental Disorders (NDDs), and those without NDDs. (Bonsu et al., 2021; Sahni et al., 2020). Understanding parental uptake of the COVID-19 vaccine provides insights into broader patterns of vaccine hesitancy within this group. Exploring this helps identify barriers and facilitators to vaccine acceptance among parents of children with ASD and other NDDs.

In a study conducted by Sahni et al., vaccine hesitancy was found to be 29.5% among parents of children with ASD, compared to 8.6% among parents of children with rheumatological conditions (Sahni et al., 2020). In another study conducted Bonsu et al., vaccine hestiancy was reported at 27.9% among parents of children with ASD and 9.5% among parents of children without ASD (Bonsu et al., 2021). Additionally, approximately 20% of parents with children who have ASD believe that vaccines are a cause of autism, a rate higher than that of control groups. This suggests that these families may experience greater levels of vaccine hesitancy (Sahni et al., 2020; Goin-Kochel et al., 2020).

Furthermore, there is a lack of research on this topic, and studies are predominantly conducted in Western countries. Given the complex nature of decision- making concerning vaccines, it is essential to conduct studies in various geographical locations and within diverse culture contexts (Dubé et al., 2014).

While two studies focusing on the parents of children with ASD and their vaccination attitudes are available in Turkey, it is essential to expand this research further (Bayturan & Celasin, 2022; Bağ & Güney, 2023).

Given the nature of ASD, coping with stress is a significant concern for parents of children with ASD, and the precise impact of this stress on both parents and their children remains unclear (Ghanouni & Hood, 2021; Vernhet et al., 2019). Gavaruzzi et al. highlighted the importance of parents managing their emotions for vaccine acceptance (Gavaruzzi et al., 2021). Parents’ responsibility to make the best decisions for their children can cause tension, especially with new parenting styles, complex information, and fear of harm. The lack of communication about childhood vaccination contributes to parental anxiety, even among those compliant with vaccination policies (Kuan et al. 2022). This stress can lead to anxiety and decision-making difficulties, contributing to vaccine hesitancy (Rodriguez et al., 2023; Shilubane & Mazibuko, 2020).

It is known that the stress and coping mechanisms of parents with children who have ASD differ from those with children who have other developmental delays or behavioral issues (Sartor et al., 2023; Lai et al., 2015). In a qualitative study, five main coping themes were identified among parents of children with ASD: lack of knowledge, cultural beliefs, prayer, strong support system, and acceptance (Shilubane & Mazibuko, 2020). Marcinechová et al. reported that parents of children with ASD used guilt, shame, and self-forgiveness more than parents of typically developing children. They often felt shame about their children’s behavior or being misunderstood by society, while parents of typically developing children felt shame about their parenting (Marcinechová et al., 2023). These studies suggest that the coping methods of parents of children with ASD may differ significantly from those of parents of children with other disorders.

While it is unclear whether coping mechanisms directly contribute to vaccine hesitancy or which specific mechanisms are involved, coping strategies may play a role in vaccine hesitancy by reducing stressors and emotional responses. It is also believed that stress and coping mechanisms can be related to decision-making processes (Palamarchuk & Vaillancourt, 2021). Families of children with ASD experience higher stress levels. Sartor et al. define parental stress as the stress resulting from a mismatch between parental demands and resources. Parents of children with ASD face numerous demands regarding their child’s behaviors, environmental adjustment problems, and guilt over not being able to devote appropriate time to the child, parental stress is higher in parents of chıldren with ASD (Sartor et al., 2023). This is the context in which we use ‘stress’ in this article.

Regarding the relationship between coping mechanisms and stress, studies show that parents of children with ASD who use dysfunctional behaviors such as active avoidance of problems exhibit higher levels of stress (Tröster & Lange, 2019; Hastings et al., 2005), whereas active coping has been shown to be a stress-reducing strategy among parents of children with ASD (Wang et al., 2013). Behavioral and cognitive responses to stress, such as avoidance, seeking social support, and information-seeking, can impact vaccine decisions (Morstead et al., 2022; Wang & Zhang, 2021). Therefore, providing accurate and reliable information about vaccines and assisting families in developing effective coping strategies could help mitigate vaccine hesitancy.

After extensive literature searches, we did not find any published articles describing the structure of coping strategies adopted by parents of children with autism and vaccine hesitancy. The aim of this study is to compare the levels of vaccine hesitancy and vaccination behaviors among parents of children with ASD, other NDDs, and those without NDDs. Additionally, the study aims to explore the relationship between stress coping mechanisms and vaccine hesitancy, and to describe and compare these coping mechanisms across the different diagnostic groups.

## Methods

### Ethical Approval

IRB approval for the study was procured from the Ethics Committee of xxx University [31.10.2023-E.300127]. Participants were informed about the study, and their consent was obtained prior to participation. All of the study procedures were in accordance with the Declaration of Helsinki and local laws and regulations.

### Study Design and Population

This cross-sectional study was conducted in xxx, one of Turkiye’s cities with a population of over one million. The study population comprises parents of children with ASD, parents of children with non-ASD-NDD, and parents of children without any NDD. The total sample size was determined to be 252 individuals, considering a significance level (α) of 0.05, a power of 0.95, and a medium effect size for vaccine hesitancy based on the literature (Bonsu et al., 2021; Goin-Kochel et al., 2020). Allowing for a potential data loss of 10%, the target was to reach a minimum of 280 participants.

Children with ASD and non-ASD-NDD were selected from four accessible special education centers within the same city. Attempts were made to reach out to parents of all children registered in these centers (total number of children with ASD: 150, children without ASD: 200), ensuring that the minimum calculated sample size was met. From November 2023 to January 2024, parents routinely applying to the centers were invited to participate, while others were contacted and invited by a familiar institution employee. The inclusion criteria for participation in the study were set as having at least one child with ASD or other NDD registered in these institutions and being literate. Volunteers meeting these criteria were identified. Parents of children without NDD were selected by aiming to reach all children (*n* = 250) admitted to the general pediatrics service of a hospital in the same region during November 2023 for various acute illnesses or medical conditions. The inclusion criterion for this group also included the absence of a coexisting NDD diagnosis accompanying the child’s illness.

### Survey Instruments and Data Collection

Survey prepared by the researchers, based on the literature and aligned with the study’s objectives, were used to inquire about parents’ socio-demographic status (age, gender, marital status, education level, family monthly income), their and their children’s vaccination statuses (whether they received the COVID-19 vaccine, the status of the child’s regular vaccinations in the routine immunization schedule, and if applicable, the status of the child’s younger sibling’s regular vaccinations in the immunization schedule), and the child’s general characteristics (age, gender, number of siblings, perceived cause of the illness).

For the purposes of this study, vaccine hesitancy is defined as the delay in acceptance or refusal of vaccines despite the availability of vaccination services. It encompasses concerns about vaccine safety, efficacy, and necessity. The ‘Vaccine Hesitancy Scale in Turkish’ (VHLS), developed by Kilincarslan et al., was utilized to measure participants’ levels of vaccine hesitancy. This scale was chosen due to its robust theoretical foundation, high validity, and reliability in the Turkish context. The VHLS is designed to measure general vaccine hesitancy, not specifically parental vaccine hesitancy towards childhood vaccines. It consists of 21 questions and four factors, covering different aspects of vaccine hesitancy.

Benefit and protective value of vaccine, this subscale consists of 5 questions, scored between 5 and 25. It includes questions about the protective effects of vaccines against diseases. Vaccine repugnance, this subscale consists of 6 questions, scored between 6 and 30. It includes questions related to the side effects and perceived harms of vaccines. Solutions of non-vaccination, this subscale consists of 5 questions, scored between 5 and 25. It includes questions about the preference for natural immunity over vaccination and the compulsion of vaccination. Legitimization of vaccination, this subscale consists of 5 questions, scored between 5 and 25. It includes questions about whether vaccination is deemed unnecessary due to the low prevalence of infectious diseases or because other children are already vaccinated.

The total score of the scale can range from 21 to 105 (with 21 questions scored between 1 and 5), where a higher score indicates higher vaccine hesitancy. The scale demonstrated a high Cronbach’s alpha of 0.905, indicating excellent internal consistency. Using this scale allows for a comprehensive understanding of the multifaceted nature of vaccine hesitancy among the study population (Kilincarslan et al. 2020).

The Coping Style Scale Brief Form (CSS-BF), created by Carver in 1997, was selected for its extensive validation across multiple languages and its comprehensive coverage of different coping mechanisms. This scale is frequently used to assess mechanisms for coping with daily stressful situations as well as highly stressful health-related situations (such as a child’s illness) (Carver, 1997; Bacanli et al. 2013). The Turkish validation of this 28-item scale with 14 dimensions was conducted by Bacanli et al. Each of the 14 coping mechanisms (using instrumental social support, humor, focus on and venting emotions, substance use, acceptance, suppression of competing activities, turning to religion, denial, behavioral disengagement, mental disengagement, restraint coping, positive reinterpretation, using emotional social support, and planning) is assessed through two questions on 4-point Likert scale, making a total of 28 questions. A higher score indicates a more frequent use of that mechanism (Bacanli et al. 2013).

Participants were provided with a brief explanation before filling out the surveys, and a conducive environment was ensured for them to answer the questions independently. The surveys were administered in person. On average, it took approximately 20 min to complete the surveys.

### Data Analysis

The demographic characteristics of the participants are presented as the mean and standard deviation or as frequencies and percentages. Descriptive characteristics were provided by stratifying children into ASD, non-ASD NDD, and without NDD groups. As the main outcome, the vaccine hesitancy scale score was treated as a continuous variable, reflecting the inherent nature of vaccine hesitancy. Different subscale scores encompassing various coping mechanisms were calculated separately for the CSS-BF.

For the correlation of non-parametric data, the Spearman correlation test was applied, and the results were interpreted as follows: 0-0.19 as very weak, 0.20–0.39 as weak, 0.40–0.59 as moderate, 0.60–0.79 as strong, and 0.80-1.00 as very strong. In the univariate analyses, either ANOVA or chi-square tests were employed for comparing the three groups (children with ASD, non-ASD NDD, and without NDD), and odds ratios were also provided for categorical data. In the evaluation of statistically significant results among the three groups, an appropriate post-hoc test was applied with Bonferroni correction.

For multivariate analysis, individuals with a VHS total scorer above the overall median value were classified as having high vaccine hesitancy, while those with a score at or below the median were classified as having low vaccine hesitancy. Additionally, the parent’s COVID-19 vaccine status, the child’s vaccine status, and the vaccine status of the child’s sibling were examined as categorical variables. In multivariate analyses, adjustments were made according to the variables specified in the model, and logistic regression was applied. Model results were summarized by odds ratios with 95% confidence intervals, and statistical significance for coefficients of the multiple regression model was assessed at the 0.05 level. Analyses were conducted using SPSS (Statistical Package for Social Science) 29.

## Results

### Characteristics by Participants

One parent for each of 299 children were included in the study: 85/150 (56.7%) diagnosed with ASD, 115/200 (57.5%) with non-ASD NDD, and 99/250 (39.6) without NDD. Among children with non-ASD NDD, the most common diagnoses were intellectual disability (*n* = 35/115, 30.4%), specific learning disorder (*n* = 28/115, 24.3%), and communication disorders (*n* = 15/115, 13%). Among the parents, the mean age was 36.5 ± 6.8 years. Of the parents surveyed, 251 of them (83.9%) were female. Detailed demographics and characteristics of the study participants are outlined in Table 1. When individuals excluded from the analysis due to missing data were compared with those included in the analyses based on their socio-demographic characteristics, it was found that 13/14 (92.9%) individuals removed from the scale scoring, versus 100/285 (35.1%) individuals who completed the scale, had education levels of primary school or below (*p* < 0.001). Furthermore, among the 6/14 (42.9%) individuals excluded from versus 48/285 (16.8%) respondents reported having a low family income (*p* = 0.016). For other variables, no statistically significant difference in socio-demographic characteristics was detected between individuals with missing data and those who responded (p values > 0.05).

**Table 1:** General characteristics of the participants according to diagnostic groups

Characteristics of Participants	Dianostic Status			*p* value
	No neurodevelopmental disorders (*n* = 99)	ASD (*n* = 85)	Non-ASD neurodevelopmental disorders (*n* = 115)	
Parent’s sex (*Female*), n (%)	80 (80.8)	72 (84.7)	99 (86.1)	0.562
Parent’s age, mean ± std.dev	36.5 ± 5.9	36.3 ± 6.6	36.8 ± 7.6	0.860
Parent’s marital status (*Married*), n (%)	93 (93.9)	83 (97.6)	106 (92.2)	0.250
Parent’s education level				< 0.001
*Primary school and below*	22 (22.1)	37 (43.5)	54 (47)	
*High school*	21 (21.2)	28 (32.9)	33 (28.7)	
*University level and higher*	56 (56.6)	20 (23.5)	28 (24.3)	
Family income level				< 0.001
*Low*	9 (9.1)	16 (16.5)	31 (27)	
*Middle*	47 (47.5)	55 (64.7)	76 (66.1)	
*High*	43 (43.4)	16 (18.8)	8 (7)	
Child’s age, mean ± std.dev	7 ± 3.5	7,4 ± 3.8	7,8 ± 3.6	0.254
Child’s sex (*Female*), n (%)	38 (38.4)	23 (27.1)	32 (27.8)	0.159
Number of child, mean ± std.dev	1.9 ± 0.7	2.3 ± 1.2	2.3 ± 1	0.008

ASD: Autism Spectrum Disorder

### Vaccine Hesitancy

In the assessment of the VHS score, 285/299 individuals (95.3%) who fully completed the scale were included in the analyses A statistically significant relationship was found between parents’ total vaccine hesitancy scores and all subscale scores with the children’s diagnostic groups. Parents of children with ASD have higher vaccine hesitancy scores. Detailed results are provided in Table 2. Individuals with a vaccine hesitancy score of 50 or above, the median value, were classified as having high vaccine hesitancy (*n* = 146, 51.2%), while those with a score below 50 were classified as having low vaccine hesitancy. The analysis for high vaccine hesitancy was adjusted for demographic and socioeconomic variables including the parents’ age and gender, their level of education, family income, as well as the gender and age of the child, and was examined according to the children’s diagnostic groups. The adjusted odds ratio (OR) for parents of children with ASD for high vaccine hesitancy is 2.66 (95% CI: 1.39–5.06), compared with parents of children without NDD. The OR for parents of non-ASD NDD children compared with parents of children without NDD was 1.30 (95% CI: 0.71–2.38). Detailed information is provided in Table 3.

**Table 2:** Vaccine hesitancy and vaccination status according to diagnostic groups

Vaccination status and vaccine hesitancy	Diagnostic Status			*p* value
	No neurodevelopmental disorders (*n* = 99)	ASD (*n* = 85)	Non-ASD neurodevelopmental disorders (*n* = 115)	
Not being vaccinated parents for COVID-19, n (%) (*n* = 292)	15 (15.5)	26 (30.6)	18 (16.4)	0.018
Change to not being vaccinated in sibling^1^, n (%) (*n* = 142)	6 (20.7)	14 (29.8)	13 (20.3)	0.468
Vaccine Hesitancy (total)^2^, mean ± std.dev (*n* = 285)	47.08 ± 14.50	57.36 ± 15.19	49.56 ± 15.00	< 0.001^a, b^
Vaccine Hesitancy (benefit and protective value of vaccine)^2^, mean ± std.dev (*n* = 285)	11.84 ± 4.95	14.29 ± 4.77	12.24 ± 5.06	0.002^a, b^
Vaccine Hesitancy (vaccine repugnance)^2^, mean ± std.dev (*n* = 285)	16.16 ± 5.66	19.48 ± 5.73	16.08 ± 5.26	< 0.001^a, b^
Vaccine Hesitancy (solutions for non-vaccination)^2^, mean ± std.dev (*n* = 285)	10.07 ± 4.28	13.18 ± 4.79	11.64 ± 4.68	< 0.001^a^
Vaccine Hesitancy (legitimization of vaccine hesitancy)^2^, mean ± std.dev (*n* = 285)	9.01 ± 3.6	10.41 ± 3.62	9.60 ± 3.76	0.039^a^

^a^ a statistically significant difference was detected between the group without neurodevelopmental disorders and the ASD group, ^b^ a statistically significant difference was detected between the group non-ASD neurodevelopmental disorders and the ASD group. ^1^ The child receives routine childhood vaccinations, there is a change in the vaccination status of their sibling (if applicable), indicating that the sibling is not being vaccinated.^2^ Vaccine hesitancy total score ranges from 21 to 105, benefit and protective value of vaccine from 5 to 25, vaccine repugnance from 6 to 30, solutions for non-vaccination from 5 to 25, and legitimization of vaccine hesitancy from 5 to 25

**Table 3:** Multivariate analysis of vaccine hesitancy and vaccination status according to diagnostic groups

Diagnostic Status	Not being vaccinated parents for COVID-19 (*n* = 292)		Change to not being vaccinated in sibling (*n* = 142)		High Vaccine Hesitancy (*n* = 285)^1^	
	Unadjusted OR	Adjusted OR	Unadjusted OR	Adjusted OR	Unadjusted OR	Adjusted OR
No neurodevelopmental disorders (*n* = 99)	Ref.		Ref.		Ref.	
ASD (*n* = 85)	2.41 (1.18–4.94)	2.57 (1.17–5.65)	1.63 (0.54–4.86)	1.40 (0.45–4.40)	2.59 (1.42–4.74)	2.66 (1.39–5.06)
Non-ASD neurodevelopmental disorders (*n* = 115)	1.07 (0.51–2.26)	1.02 (0.45–2.34)	0.98 (0.33–2.89)	1.07 (0.34–3.35)	1.34 (0.77–2.33)	1.30 (0.71–2.38)

* Adjusted odds ratios (ORs) have been adjusted for the parent’s age, gender, level of education, the family’s income, and the child’s gender and age

ASD: Autism Spectrum Disorder, OR: Odds Raito, ^1^ Of the 285 individuals with a vaccine hesitancy score, 146 were classified as having high vaccine hesitancy based on a median score of 50. In the reference group, 41 individuals (41.4%) were considered to have high vaccine hesitancy, in the ASD group 54 individuals (63.5%), and in the non-ASD neurodevelopmental disorders group 51 individuals (44.3%) were found to have high vaccine hesitancy

### Vaccine Behaviour

In the assessment of vaccination behaviors, 292/299 individuals (97.3%) who reported their COVID-19 vaccination status for analyses related to COVID-19 vaccine uptake, and 142/146 children (97.3%) who have a younger sibling and whose vaccination status was available, were included in the respective analyses.

A statistically significant difference was found in the relationship between parents not receiving the COVID-19 vaccine and the children’s diagnostic groups (*p* = 0.018). However, no statistically significant relationship was found between the vaccination of siblings in families with routinely vaccinated children and the children’s diagnostic groups (*p* = 0.468). Detailed information is provided in Table 2. The adjusted odds ratio (OR) for parents of children with ASD not being vaccinated for COVID-19 is 2.57 (95% CI: 1.17–5.65), compared with parents of children without NDD. The OR for parents of non-ASD NDD children compared with parents of children without NDD was 1.02 (95% CI: 0.45–2.34). When a child receives routine childhood vaccinations, the OR indicating that the sibling is not being vaccinated is 1.40 (95% CI: 0.45–4.40). The OR for parents of non-ASD NDD children compared with parents of children without NDD was 1.07 (95% CI: 0.34–3.35). Detailed information is provided in Table 3.

### Coping Styles

In the analysis of the association between diagnostic groups and the subscales of the coping styles scale, significant differences were identified in humor, turning to religion, and positive reinterpretation (*p* = 0.002, 0.036, and 0.035, respectively). Subsequent post hoc analyses determined that the scores for humor were significantly lower in the ASD group than in the group without DD (*p* = 0.002). Furthermore, the scores for positive reinterpretation were found to be significantly higher in the group without NDD compared to those in the non-ASD NDD group (*p* = 0.035). In the parents of children in the without NDD diagnostic group, a statistically significant weak correlation was observed between VHLS total score and the substance use coping style (rho = 0.226, *p* = 0.008). In the same group, the substance use coping style was also found to have a weak correlation with the subscale scores for benefit and protective value of the vaccine and legitimization of vaccine hesitancy (rho = 0.266; 0.298 respectively). In the parents of children with ASD, a statistically significant weak positive correlation was found between VHLS total score and restrain coping style (respectively, rho = 0.280, *p* = 0.010). Parents of children with ASD also showed statistically significant weak correlations between the restrain coping style and the subscale scores for vaccine repugnance, solutions for non-vaccination, and legitimization of vaccine hesitancy (rho = 0.271; 0.256; 0.258 respectively). In the same group, statistically significant weak correlations were also found between the focus on and venting of emotions coping style and the subscale scores for benefit and protective value of the vaccine, solutions for non-vaccination, and vaccine hesitancy (rho = 0.240, 0.246, respectively). In the parents of children in the non-ASD NDD diagnostic group, a statistically significant weak correlation was identified between VHLS total score and denial coping style (rho = 0.224, *p* = 0.024). In the same group, statistically significant weak correlations were found between the denial coping style and the subscale scores for vaccine repugnance and solutions for non-vaccination (rho = 0.249, 0.213, respectively). Detailed information is provided in Table 4.

**Table 4:** Correlations between coping styles and VHLS subscales across different diagnostic groups

Diagnostic Group	Coping Style	VHLS Score	Correlation coefficient
No neurodevelopmental disorders	Substance use	VHLS total score	0.226
		Benefit and protective value	0.266
		Legitimization of vaccine hesitancy	0.298
Autism Spectrum Disorder	Restrain	VHLS total score	0.280
		Vaccine repugnance	0.271
		Solutions for non-vaccination	0.256
		Legitimization of vaccine hesitancy	0.258
	Focus on and venting of emotions	Benefit and protective value	0.240
		Solutions for non-vaccination	0.246
Non-ASD neurodevelopmental disorders	Denial	VHLS total score	0.224
		Vaccine repugnance	0.249
		Solutions for non-vaccination	0.213

VHLS: Vaccine Hesitancy Literacy Scale

## Discussion

Our study shows that parents of children with ASD exhibit more vaccine hesitancy than parents of children with other diagnostic groups. This finding is consistent with previous research, such as a 2017 study in Texas involving 332 children, which showed that parents of children with ASD were more hesitant about vaccines than those of children with non-ASD-NDD or rheumatologic conditions, as well as the general pediatric population where vaccine hesitancy was measured using the Parent Attitudes About Childhood Vaccines (PACV) scale (Sahni et al., 2020). Another study from the same year in Texas also observed increased vaccine hesitancy in the ASD group, although the results were not statistically significant where vaccine hesitancy was measured using the PACV scale (Bonsu et al., 2021). A more recent study from Qatar in 2022 supported these findings, showing that 18.2% of the ASD group exhibited vaccine hesitancy, compared to 11.7% in the control group where vaccine hesitancy was measured using the PACV scale (Khoodoruth et al., 2023). he higher vaccine hesitancy among families of children with ASD, which we can define as delaying or refusing vaccines despite the availability of vaccination services, may be due to the perceived relationship between ASD and vaccines. Addressing this perceived link could be an important intervention point to reduce vaccine hesitancy in these families (Goin-Kochel et al., 2015, 2020; Mohamed et al., 2019; Pivetti et al., 2020; Sahni et al., 2020).

Our observations also indicate that parents of children with ASD are less likely to have received the COVID-19 vaccine themselves. For instance, a 2021 study on the intention to vaccinate against COVID-19 found that 53.8% of parents with ‘diagnosed children’, of whom 38.4% were children with ASD, intended to vaccinate, slightly lower than the 55.6% intention observed among parents of healthy children (Al Saad et al., 2023). Similarly, in Qatar, the percentage of children with ASD who were vaccinated for COVID-19 stood at 24.3%, which is lower than the 27.8% observed in the control group. The willingness to vaccinate for COID-19 was 35.7% among the ASD group compared to 42% among the control group (Khoodoruth et al., 2023). Furthermore, a 2021 study in New York State involving 393 children with developmental delays found that families of children with autism were significantly less inclined to vaccinate for COVID-19 their children compared to families of children with other diagnoses (Bonuck et al., 2021). While previous studies have primarily focused on vaccination intentions during the early stages of vaccine rollout, our study, conducted later, inquires about the actual vaccination status, offering a different perspective. Our study broadens the scope of investigation to encompass not only children’s vaccination status but also that of the parents themselves. It demonstrates an adjusted odds ratio of 2.57 (95% CI: 1.17–5.65) for the likelihood of parents in the ASD group not getting vaccinated. This indicates a higher risk compared to what has previously been documented in the literature. The quick development and emergency use of COVID-19 vaccines have raised unique safety and efficacy concerns. This differs from hesitancy toward established vaccines. Understanding these differences is key to creating effective interventions (Bianchi 2023).

Additionally, our analysis revealed notable observations regarding the vaccination of younger siblings in families with children diagnosed with ASD. While not reaching statistical significance, the odds ratio of 1.40 (95% CI: 0.45–4.40) for these siblings not being vaccinated—despite the index child receiving routine vaccinations— indicates a possible trend of hesitancy or refusal.This observation is in line with a comprehensive study conducted in the USA, which included 3,729 children with ASD and 592,907 without, revealing that the adjusted relative risk for full vaccination among siblings in the ASD group varied between 0.86 and 0.96, influenced by age groups (Zerbo et al., 2018). This finding is further supported by research from Turkey, where two separate studies consistently reported a higher incidence of vaccine refusal among siblings of children with ASD (Bag and Guney 2023; Bayturan & Celasin, 2022).

Furthermore, our investigation into the coping styles of parents with children diagnosed with ASD uncovers an area not widely covered in existing literature. Although many studies have examined the coping mechanisms employed by these parents (Clifford & Minnes, 2013; Lyons et al., 2010; Obeid & Daou, 2015), the connection between vaccine hesitancy and coping styles has not been thoroughly investigated. Our findings contribute a novel insight into this gap, identifying a statistically significant, though weak, correlation between vaccine hesitancy and the use of restraint coping style among parents of children with ASD. This specific correlation was not observed within the other diagnostic groups examined in our study.

One key consideration that led us to evaluate coping strategies is the observed link between restraint coping mechanisms and increased vaccine hesitancy, particularly during the COVID-19 pandemic. Studies have shown that individuals using negative coping mechanisms, such as avoidance, exhibit higher levels of vaccine hesitancy (Morstead et al., 2022; Wang & Zhang, 2021). It is also hypothesized that stress and coping mechanisms can be related to decision-making processes Palamarchuk & Vaillancourt, 2021).

Stress coping, defined as behavioral and cognitive responses aimed at reducing stressors or emotional responses, could be a crucial support area for those experiencing vaccine hesitancy. Providing accurate and reliable information about vaccines and developing effective coping strategies could help mitigate vaccine hesitancy.

Considering vaccination as a proactive approach to health, there may be a link between vaccine hesitancy and the preference for passive coping strategies among parents. Yet, the exclusive detection of this correlation in the ASD group—absent in the cohorts of parents of children with non-ASD-NDD or without NDD—may indicate a distinct aspect of the vaccine decision-making process in the ASD community. This specificity suggests that vaccine hesitancy among these parents may be deeply intertwined with broader psychological and behavioral dynamics unique to the experience of raising a child with ASD. Such a finding underscores the necessity for further research to unravel these complex interactions, aiming to inform more effective communication and intervention strategies tailored to the needs and concerns of this particular parent group. These interpretations highlight the need for further empirical investigation.

This study further highlights the significant issue of vaccine hesitancy within the ASD community, not just compared to parents of neurotypical children but also those with children having non-ASD NDD. Our findings show lower vaccination levels among younger siblings and even the personal vaccination commitment of parents in the ASD group. Such results emphasize the need for targeted, detailed guidance on vaccinations for families of children with ASD. It’s important to address the hesitancy and also actively keep track of and follow up on the vaccination status of siblings and confirm the vaccination acceptance among parents themselves.

The need for interventions specifically designed to address vaccine hesitancy in the ASD community is clear. Tailored advice that meets the unique concerns and information needs of these families is crucial. A systematic method to monitor and promote vaccination among both the children and their parents is expected to improve vaccination coverage in this group. By meeting these specific needs, public health efforts can effectively overcome vaccination barriers, ensuring that families of children with ASD receive full support in making informed health decisions.

### Strengths and Limitations

The study’s limitations include its cross-sectional nature, which necessitates caution in causal interpretation. Vaccination status data, self-reported by participants, may affect information accuracy. Despite efforts to comprehensively reach eligible participants within the specified timeframe, the voluntary nature of participation might not fully represent the broader population, leaving the characteristics of non-participants undefined. Furthermore, observed discrepancies in socio-demographic characteristics between participants with complete data and those with missing information highlight the potential for bias, which could influence the generalizability of our results. Additionally, the demographic differences between groups and the unique population of hospital-admitted children for the non-NDD group should be acknowledged, as these factors may limit the comparability and generalizability of our findings.

The strengths of our study include focusing on the families of children with ASD, a significant yet relatively overlooked community in vaccine hesitancy research; offering a comprehensive perspective on vaccination that encompasses not only childhood vaccines but also the vaccination status of siblings, the COVID-19 vaccination status of parents, and vaccine hesitancy levels, thus providing a broad view on vaccine-related attitudes; collecting data through direct interviews with families, overcoming potential challenges in reaching families of children with ASD, and achieving a relatively high sample size; conducting the study in a region with limited research on culturally variable topics such as vaccine hesitancy in ASD; and examining the relationship between coping styles and vaccine hesitancy among families of children with ASD.

## Conclusion

Despite robust scientific evidence refuting any link between vaccines and ASD, vaccine hesitancy among parents of children with ASD remains a significant challenge. Addressing this issue is crucial for public health, necessitating targeted educational initiatives and continuous monitoring of these families. The enduring nature of vaccine hesitancy, despite clear scientific disproof, calls for comprehensive research to uncover the multifaceted reasons behind this reluctance. Implementing specific interventions, perhaps starting with community-based educational programs and support groups facilitated by healthcare providers, and exploring the relationship between parental coping styles and vaccine attitudes, are critical steps forward. Collaborative efforts involving a spectrum of healthcare professionals and community leaders are essential in devising and executing strategies that effectively mitigate hesitancy and promote vaccination among this vulnerable group.
